# Head injury/traumatic brain injury and the risk of dementia: An observational and Mendelian randomization study

**DOI:** 10.1016/j.tjpad.2025.100468

**Published:** 2026-01-08

**Authors:** Ziyu Ouyang, Bin Jiao, Xuewen Xiao, Qijie Yang, Yuan Zhu, Lu Shen, Nan Li

**Affiliations:** a Department of Neurology, Xiangya Hospital, Central South University, Changsha, Hunan, China; b Bioinformatics Center & National Clinical Research Centre for Geriatric Disorders, Xiangya Hospital, Central South University, China; c Engineering Research Center of Hunan Province in Cognitive Impairment Disorders, Central South University, Changsha, China; d Hunan International Scientific and Technological Cooperation Base of Neurodegenerative and Neurogenetic Diseases, Changsha, China; e Key Laboratory of Hunan Province in Neurodegenerative Disorders, Central South University, Changsha, Hunan, China; f Key Laboratory of Organ Injury, Aging and Regenerative Medicine of Hunan Province, Changsha, Hunan, China

**Keywords:** Head injury, Traumatic brain injury, Dementia, MRI, Mendelian randomization

## Abstract

**Background:**

This study aimed to investigate the link between head injury (HI)/traumatic brain injury (TBI) and dementia risk, as it remains unclear.

**Methods:**

We examined the associations between HI/TBI-related factors, including the frequency of HIs and the severity of TBI, and the risk of dementia (*n* = 397,581), as well as neuroimaging outcomes (*n* = 42,380) using prospective data (50 years at baseline) from the UK Biobank. In the observational analyses, Cox proportional-hazards modeling and logistic regression were used to estimate the associations between factors. Mendelian randomization (MR) was conducted to investigate the underlying causality between TBI (*n* = 392,423, n_cases_=19,842) and Alzheimer's disease (AD) (*n* = 41,944, n_cases_=21,982).

**Results:**

During the 12.5-year follow-up period, 7524 participants developed dementia. HI and TBI conferred an increased dementia risk (hazard ratio (HR)=1.72, 95 % confidence interval (CI): 1.50–1.97; HR=1.86, 95 % CI: 1.46–2.38, respectively). The risk increased in relation to recurrent HIs (HR=4.05, 95 % CI: 2.24–7.32) or severe TBI (HR=4.50, 95 % CI: 3.18–6.37). Dementia risk was highest during the first 30 months following HI occurrence (HR=2.20, 95 % CI: 1.66–2.92), whereas there was no association after 40 years post-HI. Patients with recurrent HIs also exhibited reduced hippocampal volumes and increased white matter hyperintensity. HI was additionally associated with poorer reasoning ability and longer reaction time. Besides, the MR analysis supported a causal association between TBI and AD (odds ratio (OR)=1.17, 95 % CI: 1.01–1.37).

**Conclusion:**

These results imply that HI/TBI is associated with increased dementia risk. Strategies are needed to mitigate the impact of subsequent dementia.

## 1 Introduction

Head injury (HI) is a significant public health concern. In the United States alone, the prevalence of self-reported HIs among individuals aged 40 years and older was shown to be 157 per 1000 [1]. Traumatic brain injury (TBI) is a subtype of HI and is precisely identified as "an alteration in brain function, or other evidence of brain pathology, caused by an external force"[2]. The age-standardized incidence rate for TBI was shown to be 346 per 100,000 individuals in the population [3]. Increases in population density and the use of motor vehicles, motorcycles, and bicycles may increase the incidence of HI and TBI. Dementia, which could be related to such injury, is becoming an increasingly prevalent problem, resulting in a high disease burden. With an aging and expanding population, the number of people with dementia is expected to increase from 57.4 million cases in 2010 to 152.8 million cases in 2050 [4]. The etiology of dementia is multifactorial; however, modifiable risk factors account for approximately 40 % of all cases [5].

In the past 10 years, there has been an increasing focus on the long-term damage to the nervous system caused by HI/TBI. However, limited studies have examined the association between HI/TBI and dementia risk, and the results of those that have were inconclusive. For instance, prospective studies conducted in Denmark, Sweden, the USA and the UK have reported higher rates of dementia among people with a history of HI/TBI [6], [7], [8], [9], [10], [11], although these studies failed to account for certain confounding factors at baseline (e.g., the degree of physical activity and the presence of sleep disorders), and there were risks of reverse causality[12]. In addition, two prospective studies involving analyses from postmortem autopsies have shown that TBI may not be involved in the neuropathology of dementia [[13], [14], [15]]. It is important to clarify the relationship between HI/TBI and dementia to facilitate early cognitive intervention in patients experiencing HI/TBI. One means of improving the quality of the data analysis would be through the use of Mendelian randomization (MR), which is an epidemiological strategy that utilizes genetic variants associated with exposure to evaluate causal effects on study outcomes [16]. To date, no study has employed MR analysis to investigate the association between TBI and Alzheimer’s disease (AD).

In this study, we aimed to use data collected from the UK Biobank to construct a large-scale sample cohort of patients with complete baseline information to examine the association of HI/TBI with the incidence of dementia and to explore the underlying pathways by investigating structural brain changes using magnetic resonance imaging (MRI). A MR analysis was conducted to demonstrate these associations were causally related.

## 2 Materials/Subjects and methods

### 2.1 Study samples

The data analyzed in this study were collected from the United Kingdom (UK) Biobank, a major biobank containing information from over 50,000 persons aged 40–69 years. This study recruited adults aged ≥ 50 years whose data were collected over the course of four examinations, during which time baseline parameters were examined frequently. Field IDs of UK biobank used in the analyses are provided in Supplementary **Table S1**. Patients diagnosed with dementia before recruitment were excluded (Supplementary **Figure S1**). Based on the number of participants for whom data were available, the sample sizes ranged from approximately 40,000 for the neuroimaging outcomes to over 400,000 for the incidence of dementia. The National Health Service (NHS) National Research Ethics Service was consulted for clearance to conduct the study, and every participant provided informed consent for inclusion.

### 2.2 Exposure

Both HI and TBI were included as exposures in this study. We gathered data on HI from the NHS electronic health records (EHR) that were connected to the UK Biobank database and merged them with information based on self-reported medical conditions from site interviews. The EHR contain information on hospital admissions since 1980. The earliest date of a self-reported HI was tracked back to 1939. TBI is a subtype of HI and was diagnosed according to hospital inpatient and primary care records. The date of diagnosis was used to record the earliest HI event. Patients with TBI were further classified into mild TBI (mTBI) and severe TBI groups, with mTBI mainly involving concussion, whereas severe TBI involved diffuse brain injury, focal brain injury, traumatic subdural hemorrhage, and traumatic subarachnoid hemorrhage. Hospital admission and primary care diagnoses were determined using the International Classification of Diseases (ICD) 9/10 codes. In addition, the diagnostic codes for HI and its subtypes are provided in Supplementary **Table S2**.

### 2.3 Primary outcome

The UK Biobank defines dementia cases using an algorithm that is based on self-reported disease data and the linked data of hospital admission and death registries based on ICD9/10 codes. Dementia cases were mainly reported by hospital specialists, general practitioners in primary care, or by staff from the UK death registry system. However, the UKB dataset does not include detailed diagnostic data such as cerebrospinal fluid examination or neuroimaging findings.

The earliest recorded code was used (updated as of Jan. 2022). Participant years were determined from the date of enrolment to the date of the censoring event (i.e., dementia diagnosis, death, or loss to follow-up). The end of follow-up was defined as November 12, 2021, the date at which the final endpoint event occurred.

### 2.4 Neuroimaging outcomes

The total brain volume, and the volumes of the gray matter, white matter, and hippocampi, as well as the white matter hyperintensity (WMH) values were used. All imaging values, except for the WMH, were normalized based on head size. The data were ascertained using T1-structural brain MRI and T2 fluid-attenuated inversion recovery (FLAIR) brain scans conducted by the UK Biobank's imaging staff. T1 images were processed with FreeSurfer; surface templates were used to extract imaging-derived phenotypes referred to as atlas regions’ volume. FreeSurfer FAST (ID 1101) atlases corresponding to 66 cortical and subcortical regions were used in this study.

### 2.5 Cognitive function outcomes

We selected seven cognitive tests administered during the UK Biobank imaging assessment to evaluate distinct cognitive domains. These measures included trail-making tests A and B, reaction time, numeric memory, reasoning, symbol digit substitution, and pairs matching.

### 2.6 Covariates

The following covariates were included in our study: age, sex, race, and apolipoprotein E ε4 allele (*APOE*-ε4) status, as well as the Townsend Deprivation Index, level of education, income, alcohol consumption per week, physical activity, medical comorbidities, psychiatric comorbidities, smoking status, body mass index (BMI), sleep disorders, and hearing difficulties. Details of the covariate categorizations are provided in Supplementary **Table S3**. Notably, sleep issues, psychiatric comorbidities, and hearing problems may be downstream consequences of HI/TBI and may be potential mediators of the association between HI/TBI and dementia [17,18].

### 2.7 Two-Sample MR analysis

We performed a series of MR analyses to further explore whether causal associations exist between HI and dementia-related outcomes. VD and ACD were not included in the MR analysis simply because no publicly available summary data yet exist for these dementia endpoints. We utilized publicly available summaries of genome-wide association studies (GWAS) from the FinnGen and International Genomics of Alzheimer’s Project (IGAP) consortia. FinnGen consortium summary data were used to obtain the genetic associations of TBI, including the phenotype data of 392,423 Finns (https://www.finngen.fi). The total number of TBI cases was 19,842. Genetic associations with AD were obtained from a GWAS of patients with clinically diagnosed AD from the IGAP based on 21,982 cases and 41,944 controls of European ancestry.

### 2.8 Instrumental variable selection

We selected single-nucleotide polymorphisms (SNPs) with genome-wide significance at a threshold of *p* < 5 × 10^-6^. All SNPs were in dependent linkage equilibrium and within genomic regions (r^2^ < 0.01 and clump distance >5 kb in European populations). Sixteen SNPs were identified in this MR study.

### 2.9 Statistical analysis

Three regression models were developed for all cohort studies. Model 1 simply accounted for age and sex, whereas Model 2 accounted for confounding variables such as race, APOE-ε4 status, the Townsend Deprivation Index, level of education, income, alcohol intake, degree of physical activity, presence of medical comorbidities, smoking status, and BMI. Model 3 also included variables that could act as mediators, such as the presence of sleep disorders, psychiatric comorbidities, and hearing difficulties. The results of Model 3 should be interpreted with caution, as they may be overcontrolled and conditioned on a confounder-mediator [19]. In the analysis of ACD and some VD cases, we also included age squared to suit the fact that dementia diagnoses accelerate with increasing age. We performed two sex-, age- and *APOE-ε4*-stratified subgroup analyses, considering that there are sex, age and *APOE-ε4* differences in terms of HI and dementia risk. Age was categorized as either younger than 65 years or 65 years and older. We excluded one participant who was diagnosed with dementia two years after sustaining an HI to avoid the possibility of reverse causality. The rate of missing values ranged from 0 % (for sex and age) to 24 % (for alcohol consumption); related details are provided in Supplementary **Table S4**. We applied multiple imputations with chained equations to create multiple predictions for the missing values based on the distribution of all variables, including the exposure, outcomes, and covariates.

Using time from enrolment as the timescale, Cox proportional hazard models were used to predict the probability of developing dementia in individuals with a history of HI/TBI, relative to the probability in those without HI/TBI. The relationships between HI/TBI history and cognitive and imaging volumetric markers were evaluated using multiple linear regression models. Prior to statistical analysis, all neuroimaging measures and cognitive test scores were normalized using Z-score transformation.

In the MR analysis, the relationship of each SNP with the exposures was weighted according to its correlation with the outcomes, and estimates were merged using the inverse-variance weighting (IVW) approach. Multiple sensitivity assessments were conducted, including the following: (a) if at least half of the SNPs were valid, the weighted median method provided accurate estimates; (b) MR-Egger regression was used to detect pleiotropy and correct for it; (c) leave-one-out analysis was conducted, in which SNPs were eliminated one-by-one to determine the influence of outlier SNPs; and (d) individual Wald ratios for each SNP were displayed on funnel plots, with asymmetry indicating horizontal pleiotropy in a particular direction.

Log-minus-log plots were used to verify the proportional hazards assumption. All tests were two-tailed, with a cut-off for statistical significance set at *p* < 0.05. Statistical analyses were conducted using R Version 4.2.1.

## 3 Results

### 3.1 Characteristics of the participants

The two cohorts were identified for different purposes. We studied the risk of dementia in individuals with a history of HI/TBI compared with the same risk in those without HI/TBI by following a cohort of 397,581 eligible participants over a mean period of 12.5 years. We also defined a cohort of 42,380 eligible participants who had undergone MRI scans. In the primary sample, 7524 individuals (1.89 %) were diagnosed with ACD. There was no variation in the distributions of the covariates among the three groups (Supplementary **Table S5** and **S6**). Patients with dementia were more likely to be male than female (53 % versus 46 %, respectively); they were more likely than those without dementia to be less educated (college degree or higher of 34 % versus 47 %, respectively), to have a lower income (46 % making less than £18,000 versus 25 %, respectively), and to belong to a lower social class (Townsend Deprivation Index of −0.95 versus −1.44, respectively). At enrolment, 6179 (1.55 %) individuals in the primary study cohort had a history of HI.

### 3.2 Primary outcomes

**Fig. 1:**
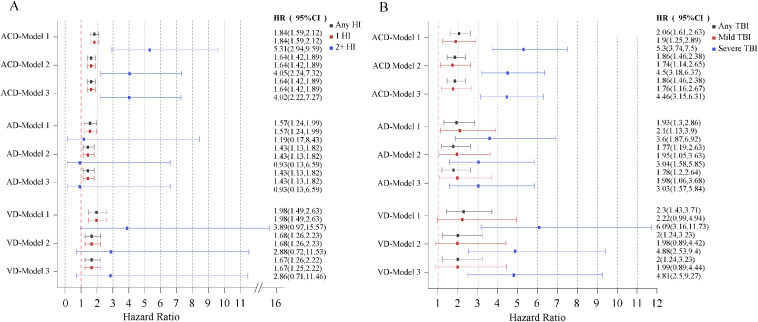
Hazard ratios for dementia, AD, and VD in association with a history of HI/TBI, the number of HI events experienced, and the severity of TBI among 397,581 UK biobank participants. a: hazard ratios for dementia, AD, and VD in association with a history of HI and the number of HI events experienced; b: hazard ratios for dementia, AD, and VD in association with a history of TBI and the severity of TBI. Model 1 is corrected for sex and age, Model 2 is adjusted for all confounders, and Model 3 is additionally adjusted for confounding mediators. Error bars indicate 95 % CIs. The null is indicated by the horizontal red dashed line, . AD, Alzheimer’s disease; VD vascular dementia; HI, head injury; TBI, traumatic brain injury; CI, confidence interval.

**Fig. 2:**
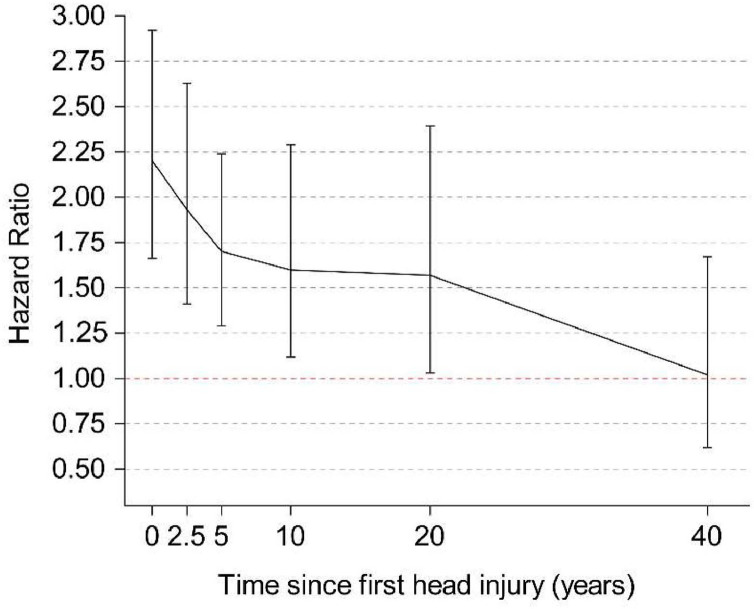
Hazard ratios for dementia according to the time since HI. Hazard ratios are based on Model 2 and are adjusted for all confounders. Error bars represent 95 % CIs. The null is indicated by the horizontal red dashed line. HI, head injury; CI, confidence interval.

Within the standard adjustment model (Model 1), we observed positive associations between HI/TBI history and all measures of dementia (Fig. 1). The associations weakened, but remained statistically significant after additional adjustments. The risk increased for participants who experienced recurrent HIs (from hazard ratio (HR)=1.64, 95 % confidence interval (CI): 1.42–1.89 for people with a single HI to HR=4.05, 95 % CI: 2.24–7.32 for people with multiple HIs) or in those with severe TBI (from HR=1.74, 95 % CI: 1.15–2.65 for people with mTBI to HR=4.50, 95 % CI: 3.18–6.37 for people with severe TBI), and mTBI was associated with both ACD (HR=1.74, 95 % CI: 1.15–2.65) and AD (HR=1.95, 95 % CI: 1.05–3.64) (Fig. 1). During the first 30 months post-HI, the risk of dementia was the highest (HR=2.20, 95 % CI: 1.66–2.92 for ACD). The risk gradually diminished at 20–40 years post-HI (HR=1.57, 95 % CI: 1.03–2.38), and no association was observed after 40 years post-HI (Fig. 2). When stratified by severity, the same trend was observed; however, the association between mTBI and ACD disappeared after 5 years post-injury (Supplementary **Table S7**). The distribution of associations differed with respect to sex, age and *APOE-ε4* (Supplementary **Tables S8-S10**). In women, a history of HI was associated with AD in model 3, whereas in men, it was associated with VD. This association was stronger in middle-aged individuals than in older people. While all *APOE-ε4* carrier states were associated with both ACD and AD, the association with AD was stronger for carriers of two alleles than for carriers of one.

### 3.3 Neuroimaging outcomes

The estimates of the associations between a history of HI and neuroimaging measures are shown in Table 1. A history of HI was associated with smaller total brain volume (*β* = −0.074, 95 % CI: −0.146 to −0.002) in Model 1, with smaller total white matter volume (*β* = −0.08, 95 % CI: −0.16 to −0.001) in Model 2, and smaller total white matter volume (*β* = −0.081, 95 % CI: −0.161 to −0.002) in Model 3. Experiencing two or more HIs was associated with smaller hippocampal volume (*β* = −0.785, 95 % CI: −1.531 to −0.039) and larger WMH volume (*β* = 0.775, 95 % CI: 0.022–1.529) in Model 1. A history of TBI and its severity stratifications were not significantly associated with neuroimaging outcomes. Notably, the association between severe TBI and total grey matter volume was close to significance (*β* = −0.248, 95 % CI: −0.541–0.045, *p* = 0.098). For women, a history of HI was associated with smaller hippocampal volume [*β* = −0.132, 95 % CI: −0.261 to −0.003 (Model 2)]. For those 65 years or older, a history of HI was associated with smaller total brain volume [*β* = −0.336, 95 % CI: −0.624 to −0.048 (Model 2)], smaller total white matter volume [*β* = −0.294, 95 % CI: −0.58 to −0.007 (Model 2)]. The amplitude and significance of the coefficient estimates for a history of HI and total white matter volume/WMH volume increased when additional adjustments for covariates were considered. For carriers of one *APOE-ε4* allele, a history of HI was associated with smaller total brain volume [*β* = −0.1, 95 % CI: −0.193 to −0.007 (Model 2)] (Supplementary **Table S8–10**). The corresponding results for the regional grey matter volumes are presented in Supplementary **Table S11**. A history of HI was correlated with reduced volumes of the globus pallidus (*β* = −0.155, *p* < 0.001), temporal pole (*β* = −0.037, *p* = 0.008), and supramarginal gyrus (*β* = −0.175, *p* = 0.017), as well as increased cerebellar volumes (Crus II: *β* = 0.010, *p* = 0.019; VIIIa: *β* = 0.017, *p* = 0.019; VIIIb: *β* = 0.001, *p* = 0.042).

**Table 1:** Linear regression estimates for HI/TBI history with total brain volume, total grey matter volume, total white matter volume, hippocampal volume, and white matter hyperintensity (WMH) Volume Among 42,380 UK biobank participants.

Outcome	Model1 beta (95 % CI)	*p*	Model2 beta (95 % CI)	*p*	Model3 beta (95 % CI)	*p*
Any HI-TBV	−0.074 (−0.146, −0.002)	0.045	−0.069 (−0.14, 0.002)	0.057	−0.07 (−0.141, 0.001)	0.052
1 HI- TBV	−0.073 (−0.145, −0.001)	0.048	−0.069 (−0.14, 0.003)	0.059	−0.07 (−0.141, 0.001)	0.055
2+ HI- TBV	−0.136 (−0.833, 0.56)	0.701	−0.099 (−0.786, 0.588)	0.777	−0.109 (−0.796, 0.578)	0.756
Any HI- TGMV	−0.038 (−0.105, 0.029)	0.262	−0.027 (−0.092, 0.039)	0.419	−0.028 (−0.094, 0.037)	0.4
1 HI- TGMV	−0.036 (−0.104, 0.031)	0.289	−0.025 (−0.091, 0.04)	0.449	−0.027 (−0.092, 0.039)	0.43
2+ HI- TGMV	−0.209 (−0.857, 0.439)	0.528	−0.168 (−0.801, 0.465)	0.603	−0.177 (−0.81, 0.456)	0.583
Any HI- TWMV	−0.075 (−0.155, 0.004)	0.064	−0.08 (−0.16, −0.001)	0.048	−0.081 (−0.161, −0.002)	0.045
1 HI- TWMV	−0.076 (−0.156, 0.004)	0.062	−0.081 (−0.161, −0.002)	0.046	−0.082 (−0.162, −0.002)	0.044
2+ HI- TWMV	0.004 (−0.766, 0.775)	0.991	0.024 (−0.744, 0.793)	0.95	0.018 (−0.751, 0.786)	0.964
Any HI-HV	−0.021 (−0.098, 0.056)	0.595	−0.007 (−0.084, 0.07)	0.858	−0.007 (−0.083, 0.07)	0.865
1 HI- HV	−0.013 (−0.09, 0.065)	0.75	0.001 (−0.077, 0.078)	0.988	0.001 (−0.076, 0.078)	0.982
2+ HI- HV	−0.785 (−1.531, −0.039)	0.039	−0.708 (−1.449, 0.034)	0.061	−0.703 (−1.444, 0.039)	0.063
Any HI-WMHV	−0.01 (−0.089, 0.07)	0.811	−0.02 (−0.098, 0.059)	0.62	−0.019 (−0.098, 0.059)	0.627
1 HI- WMHV	−0.018 (−0.098, 0.061)	0.65	−0.028 (−0.107, 0.051)	0.487	−0.028 (−0.106, 0.051)	0.492
2+ HI- WMHV	0.775 (0.022, 1.529)	0.044	0.707 (−0.036, 1.45)	0.062	0.71 (−0.033, 1.453)	0.061
TBI -TBV	−0.037 (−0.179, 0.104)	0.603	−0.025 (−0.165, 0.114)	0.722	−0.026 (−0.165, 0.114)	0.718
mTBI - TBV	−0.095 (−0.323, 0.133)	0.413	−0.076 (−0.301, 0.149)	0.51	−0.075 (−0.3, 0.15)	0.515
sTBI - TBV	−0.184 (−0.507, 0.138)	0.262	−0.194 (−0.512, 0.124)	0.232	−0.197 (−0.515, 0.121)	0.224
TBI - TGMV	−0.07 (−0.202, 0.061)	0.294	−0.052 (−0.181, 0.076)	0.424	−0.053 (−0.181, 0.076)	0.42
mTBI - TGMV	−0.096 (−0.309, 0.116)	0.373	−0.069 (−0.277, 0.138)	0.512	−0.068 (−0.276, 0.139)	0.518
sTBI - TGMV	−0.241 (−0.541, 0.059)	0.116	−0.244 (−0.538, 0.049)	0.102	−0.248 (−0.541, 0.045)	0.098
TBI - TWMV	0.013 (−0.144, 0.169)	0.875	0.013 (−0.143, 0.169)	0.87	0.013 (−0.143, 0.169)	0.871
mTBI - TWMV	−0.063 (−0.316, 0.189)	0.625	−0.06 (−0.311, 0.192)	0.643	−0.059 (−0.311, 0.193)	0.647
sTBI - TWMV	−0.059 (−0.416, 0.298)	0.745	−0.072 (−0.428, 0.284)	0.693	−0.074 (−0.43, 0.282)	0.684
TBI -HV	0.078 (−0.074, 0.23)	0.314	0.075 (−0.077, 0.226)	0.334	0.076 (−0.075, 0.227)	0.322
mTBI - HV	0.109 (−0.136, 0.353)	0.384	0.104 (−0.139, 0.347)	0.4	0.103 (−0.139, 0.346)	0.404
sTBI - HV	−0.112 (−0.464, 0.24)	0.532	−0.132 (−0.481, 0.218)	0.461	−0.13 (−0.48, 0.219)	0.465
TBI -WMHV	0.015 (−0.142, 0.173)	0.851	0.017 (−0.138, 0.172)	0.829	0.017 (−0.139, 0.172)	0.833
mTBI - WMHV	0.015 (−0.241, 0.271)	0.907	0.012 (−0.24, 0.265)	0.923	0.012 (−0.24, 0.265)	0.925
sTBI - WMHV	0.156 (−0.206, 0.519)	0.398	0.173 (−0.184, 0.531)	0.342	0.175 (−0.182, 0.532)	0.337

Model 1 is corrected for sex and age, Model 2 is adjusted for all confounders, and Model 3 is additionally adjusted for confounding mediators. Abbreviations: HI, head injury; TBI, traumatic brain injury; TBV, total brain volume; TGMV, total grey matter volume; TWMV, total white matter volume; HV, hippocampal volume; WMHV, white matter hyperintensity volume; mTBI, mild TBI; sTBI, severe TBI; CI, confidence interval.

### 3.4 Cognitive function outcomes

The associations between HI/TBI and cognitive outcomes are shown in Table 2. A history of HI was associated with longer reaction time in all adjusted models [any HI: β = 0.10, 95 % CI: 0.07–0.12; 1 HI: *β* = 0.09, 95 % CI: 0.06–0.11; 2+ HI: *β* = 0.41, 95 % CI: 0.27–0.56 (Model 2)] and with lower reasoning scores in Model 1 (any HI: *β* = −0.06, 95 % CI: −0.10 to −0.02; 1 HI: *β* = −0.06, 95 % CI: −0.10 to −0.02; 2+ HI: *β* = −0.35, 95 % CI: −0.62 to −0.10). A history of TBI was also associated with longer reaction time across all adjusted models [*β* = 0.09, 95 % CI: 0.05–0.14 (Model 2)]. Mild TBI showed no association with any cognitive score, whereas severe TBI was associated with longer reaction time [*β* = 0.29, 95 % CI: 0.18–0.39 (Model 2)] and lower reasoning scores in Model 1 (*β* = −0.23, 95 % CI: −0.39 to −0.07). In stratified analyses by sex and age, the associations between HI and both reaction time and reasoning remained consistent with the overall results (**Supplementary Tables S8** and **S9**). Notably, the association between HI and reasoning was only observed among *APOE-ε4* carriers [1 risk allele: *β* = −0.10, 95 % CI: −0.15 to −0.05; 2 risk alleles: *β* = −0.09, 95 % CI: −0.16 to −0.01 (Model 1)] (Supplementary **Table S10**), suggesting that the *APOE-ε4* genotype may mediate the relationship between HI and cognitive impairment.

**Table 2:** Linear regression estimates for HI/TBI history with cognitive function outcomes among 397,581 UK biobank participants.

Outcome	Model1 beta (95 % CI)	*p*	Model2 beta (95 % CI)	*p*	Model3 beta (95 % CI)	*p*
Any HI-Reasoning	−0.064(−0.103,−0.026)	0.001	−0.02(−0.056,0.015)	0.259	−0.02(−0.056,0.015)	0.261
1 HI- Reasoning	−0.057(−0.096,−0.019)	0.004	−0.017(−0.053,0.018)	0.342	−0.017(−0.053,0.019)	0.343
2+ HI- Reasoning	−0.36(−0.617,−0.102)	0.006	−0.153(−0.391,0.085)	0.208	−0.153(−0.391,0.086)	0.209
Any HI- RT	0.103(0.078,0.128)	<0.001	0.099(0.074,0.124)	<0.001	0.099(0.074,0.124)	<0.001
1 HI- RT	0.093(0.067,0.119)	<0.001	0.089(0.064,0.114)	<0.001	0.089(0.064,0.114)	<0.001
2+ HI- RT	0.435(0.287,0.583)	<0.001	0.413(0.27,0.557)	<0.001	0.412(0.268,0.555)	<0.001
Any HI- PM	−0.007(−0.05,0.036)	0.747	−0.001(−0.044,0.042)	0.956	−0.001(−0.044,0.042)	0.961
1 HI- PM	−0.014(−0.057,0.03)	0.534	−0.008(−0.051,0.036)	0.725	−0.008(−0.051,0.036)	0.73
2+ HI- PM	0.265(−0.013,0.542)	0.061	0.265(−0.012,0.541)	0.06	0.265(−0.011,0.542)	0.06
Any HI-NM	0.047(−0.015,0.108)	0.135	0.041(−0.018,0.101)	0.173	0.04(−0.019,0.1)	0.185
1 HI- NM	0.05(−0.012,0.112)	0.115	0.044(−0.017,0.104)	0.157	0.042(−0.018,0.103)	0.167
2+ HI- NM	−0.096(−0.514,0.322)	0.654	−0.054(−0.46,0.352)	0.793	−0.058(−0.464,0.348)	0.778
Any HI-TM1	−0.022(−0.099,0.054)	0.566	−0.004(−0.077,0.069)	0.915	−0.004(−0.077,0.069)	0.907
1 HI- TM1	−0.028(−0.105,0.049)	0.477	−0.009(−0.082,0.064)	0.809	−0.009(−0.083,0.064)	0.801
2+ HI- TM1	0.427(−0.266,1.12)	0.227	0.408(−0.252,1.069)	0.225	0.406(−0.255,1.066)	0.228
Any HI-TM2	−0.026(−0.104,0.051)	0.508	−0.009(−0.081,0.064)	0.818	−0.009(−0.081,0.064)	0.819
1 HI- TM2	−0.026(−0.104,0.052)	0.514	−0.007(−0.08,0.066)	0.85	−0.007(−0.08,0.066)	0.853
2+ HI- TM2	−0.045(−0.738,0.648)	0.898	−0.127(−0.776,0.523)	0.702	−0.133(−0.783,0.516)	0.687
Any HI-SDS	−0.025(−0.101,0.051)	0.522	−0.047(−0.116,0.022)	0.183	−0.047(−0.116,0.023)	0.186
1 HI- SDS	−0.025(−0.102,0.051)	0.517	−0.048(−0.118,0.022)	0.175	−0.048(−0.118,0.022)	0.178
2+ HI- SDS	0.015(−0.678,0.708)	0.967	0.048(−0.581,0.677)	0.882	0.048(−0.581,0.677)	0.881
TBI -Reasoning	−0.041 (−0.111, 0.03)	0.26	−0.028 (−0.093, 0.038)	0.406	−0.027 (−0.092, 0.038)	0.415
1 HI- Reasoning	0.005 (−0.107, 0.118)	0.924	0.024 (−0.08, 0.128)	0.653	0.025 (−0.079, 0.129)	0.639
2+ HI- Reasoning	−0.231 (−0.389, −0.073)	0.004	−0.163 (−0.309, −0.017)	0.029	−0.164 (−0.31, −0.018)	0.028
TBI - RT	0.08 (0.033, 0.128)	0.001	0.094 (0.048, 0.139)	<0.001	0.094 (0.049, 0.14)	<0.001
mTBI- RT	−0.015 (−0.094, 0.063)	0.701	0.007 (−0.069, 0.083)	0.856	0.007 (−0.069, 0.083)	0.849
sTBI - RT	0.3 (0.191, 0.41)	<0.001	0.287 (0.181, 0.392)	<0.001	0.287 (0.181, 0.393)	<0.001
TBI - PM	−0.016 (−0.098, 0.065)	0.695	−0.007 (−0.089, 0.074)	0.86	−0.007 (−0.089, 0.074)	0.863
mTBI- PM	−0.015 (−0.141, 0.111)	0.816	−0.006 (−0.132, 0.12)	0.926	−0.006 (−0.132, 0.12)	0.928
sTBI- PM	0.055 (−0.122, 0.231)	0.544	0.06 (−0.115, 0.236)	0.5	0.061 (−0.114, 0.237)	0.494
TBI -NM	0.035 (−0.072, 0.143)	0.521	0.04 (−0.065, 0.144)	0.455	0.039 (−0.066, 0.144)	0.464
mTBI- NM	−0.029 (−0.184, 0.126)	0.715	−0.009 (−0.16, 0.141)	0.903	−0.01 (−0.16, 0.14)	0.895
sTBI - NM	0.073 (−0.189, 0.335)	0.584	0.056 (−0.198, 0.311)	0.665	0.058 (−0.197, 0.312)	0.655
TBI -TM1	0.067 (−0.08, 0.214)	0.371	0.098 (−0.042, 0.237)	0.172	0.097 (−0.043, 0.237)	0.175
mTBI- TM1	−0.042 (−0.27, 0.186)	0.718	−0.044 (−0.261, 0.173)	0.691	−0.045 (−0.262, 0.173)	0.687
sTBI - TM1	−0.089 (−0.453, 0.275)	0.633	−0.052 (−0.398, 0.295)	0.771	−0.049 (−0.396, 0.298)	0.783
TBI -TM2	0.003 (−0.145, 0.152)	0.967	0.041 (−0.099, 0.18)	0.568	0.04 (−0.099, 0.18)	0.569
mTBI - TM2	0.122 (−0.109, 0.354)	0.3	0.132 (−0.085, 0.348)	0.233	0.133 (−0.084, 0.35)	0.229
sTBI - TM2	−0.183 (−0.561, 0.194)	0.341	−0.12 (−0.474, 0.234)	0.506	−0.115 (−0.469, 0.238)	0.523
TBI -SDS	−0.033 (−0.18, 0.115)	0.664	−0.074 (−0.208, 0.06)	0.277	−0.074 (−0.208, 0.06)	0.278
mTBI- SDS	−0.15 (−0.378, 0.078)	0.198	−0.153 (−0.36, 0.054)	0.148	−0.152 (−0.359, 0.055)	0.15
sTBI - SDS	0.026 (−0.338, 0.39)	0.889	−0.039 (−0.369, 0.292)	0.817	−0.04 (−0.371, 0.29)	0.812

Model 1 is corrected for sex and age, Model 2 is adjusted for all confounders, and Model 3 is additionally adjusted for confounding mediators. Abbreviations: HI, head injury; TBI, traumatic brain injury; RT, reaction time; PM, pairs matching; NM, numeric memory; TM, trail making; SDS, symbol digit substitution; mTBI, mild TBI; sTBI, severe TBI; CI, confidence interval.

### 3.5 Two-Sample MR analysis

**Fig. 3:**
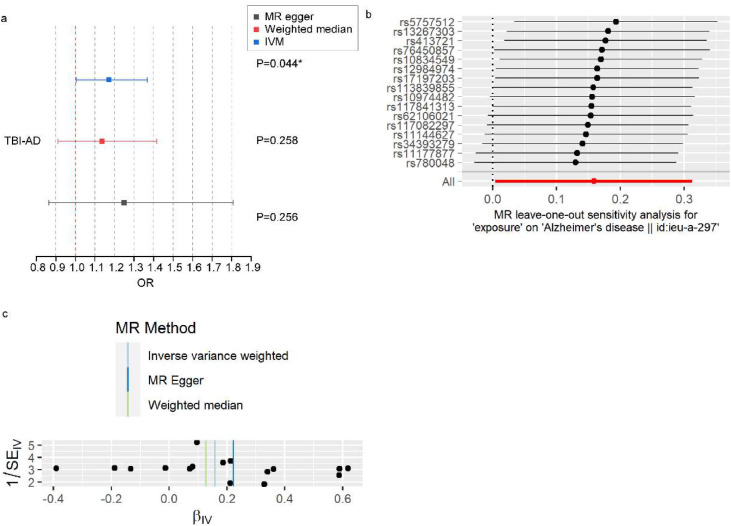
MR analysis and sensitivity analyses on the causal association between TBI and AD. a: Association of TBI History with AD at *p* < 0.05 in MR Analyses, error bars represent 95 % CIs. The null is indicated by a vertical red dashed line. b: Leave-one-out plot visualizing the causal effect of TBI on the risk of AD when leaving one SNP out. c: Funnel plots visualizing the overall heterogeneity of the MR estimates for the effect of TBI on the risk of AD. Abbreviations: MR, Mendelian randomization; TBI, traumatic brain injury; AD, Alzheimer’s disease; IVW, inverse-variance weighted; CI, confidence interval; SNP, single nucleotide polymorphism.

In the MR analysis, 16 SNPs explaining 2.06 % of the variance in TBI were used as instruments (Supplementary **Table S**12), and the mean F-statistic was 515. The estimates of the causal associations are shown in Fig. 3**a**. AD was causally affected by TBI (odds ratio (OR)=1.172, 95 %CI: 1.005–1.367) according to the IVW MR model. The effect sizes in the weighted median MR model were in the same direction as those in the IVW MR model (OR=1.136, *p* > 0.05). The MR-Egger regression revealed no evidence of directional horizontal pleiotropy (*p* > 0.05), influential SNPs, or instrument heterogeneity. In the leave-one-out analysis, we evaluated the sensitivity of the data to specific variations and found that no single instrument drove the overall effect of TBI on AD (Fig. 3**b**), and the funnel plot was symmetrical (Fig. 3**c**), indicating that none of the SNPs displayed horizontal pleiotropy. In conclusion, the MR results were determined to be accurate, and our MR analysis supports a causal association between TBI and AD.

## 4 Discussion

This large-scale cohort study examined the association between HI/TBI and dementia and found that the risk of dementia was higher in individuals with a history of HI/TBI. Additionally, the MR analysis confirmed that the relationship between TBI and AD was causal. To our knowledge, this is the first study to perform a comprehensive statistical adjustment for potential confounders that could affect the association between a history of HI/TBI and dementia, and to establish causal inference for the relationship between TBI and AD through an MR analysis. In the neuroimaging assessment, atrophy of the total brain and white matter was observed in patients with a previous HI. In addition, atrophy of the hippocampi and expansion of the WMH were observed in patients who experienced recurrent HIs.

Our findings are similar to those of six large, prospective, cohort studies that have demonstrated an association between HI/TBI and dementia[[6], [7], [8], [9], [10], [11]]. More importantly, we improved on the study design by accounting for essential confounding factors in our analysis. Comprehensive consideration of such confounders is necessary since patients with HI/TBI are more likely to be exposed to other dementia-related factors such as alcohol abuse and obesity. Such confounders were not independently quantified in previous analyses, which may have led to the miscalculation of the true prevalence of TBI-induced dementia[20]. We confirmed that HI/TBI was still associated with dementia and increased its risk, even after comprehensive consideration of the mediation effect of the confounders. After sustaining an HI, the dementia risk followed a decreasing trajectory, with the risk being the highest during the first 30 months after HI and insignificant 40 years after HI. This finding is consistent with that of a study from Denmark that reported that the risk of dementia kept decreasing after TBI[6]. The time-dependent decrease in the overall risk of dementia may reflect a combination of spontaneous recovery and neurodegeneration[21]. Our findings suggest that it is important for clinicians to measure the cognitive status of patients in the early period after HI/TBI. The reduction in the long-term impact of HI/TBI on the incidence of dementia may alleviate concerns about the neurological effects of childhood injury. This temporal pattern was consistent across severity levels; however, the effect of mild TBI diminished rapidly and became non-significant after five years, while severe TBI showed a more sustained association. These results imply that long-term dementia risk may be primarily driven by more severe injuries. However, the association between HI/TBI and dementia differed depending on the number of HIs endured, the severity, and the patient’s sex and age. For example, the risk was higher among those with recurrent HIs or severe TBI, indicating that the association between HI/TBI and dementia is dose-dependent. Studies on the association between mTBI and AD have been inconsistent[[22], [23], [24]], although the present findings demonstrate an association between mTBI and AD, proving that even mTBI increases the risk of AD. Middle-aged individuals with a history of TBI have a higher risk of dementia than the elderly, perhaps because the high prevalence of dementia among the elderly reduces the relative risk of dementia. Several studies have reported that females are also vulnerable to developing long-term dementia after sustaining a TBI, despite the neuroprotective effects of estrogen and progesterone. The present study also found that female sex is associated with different subtypes of dementia. AD disproportionately affects women, possibly due to the reduction in the levels of estrogen and progesterone, followed by the central hypopituitarism that occurs after TBI of all severities[25]. Postmenopausal women, rather than premenopausal women, experienced better outcomes than males following moderate or severe TBI, according to a previous report[26].

Similar to the results of previous studies, we found that HI is associated with accelerated atrophy of the total brain and white matter[[27], [28], [29]]. White matter atrophy may occur as a result of diffuse axonal injury, which is a common pathology among people with HI/TBI[30]. Diffuse axonal injury contributes to the pathophysiological changes that occur in dementia, such as amyloid-β accumulation[31] and tau dissociation from microtubules[32]. To the best of our knowledge, this study is the first to report that individuals who experienced recurrent HIs exhibited atrophy of the hippocampi and a larger volume of WMH. This finding provides important evidence that recurrent HIs are involved in the neuropathology of AD and VD; this is in contrast to prior studies involving postmortem autopsies that reported that TBI may not be related to the pathophysiology of AD[[13], [14], [15]]. Atrophy of the hippocampi in women partly confirmed that they are more susceptible to AD after HI/TBI, as discussed previously. Total brain and white matter atrophy is more obvious in the elderly, possibly as a result of a poorer capacity for neuronal recovery after HI/TBI[33]. In addition, we found that HI was associated with reduced grey matter volumes in the globus pallidus, temporal pole, and supramarginal gyrus, and increased cerebellar volumes. These findings are consistent with the study by Dennis et al.[34]. The reduction in cortical and subcortical grey matter volumes may reflect neuronal loss or axonal damage, whereas the cerebellar volume increase could indicate compensatory neuroplastic or neuroinflammatory processes during recovery.

Our findings are consistent with previous study[35], indicating that HI/TBI primarily affects attention and executive functions rather than memory performance. We observed a dose–response relationship, where greater frequency and severity of HI/TBI were associated with poorer cognitive outcomes. Interestingly, the association between HI and reasoning ability was only evident among *APOE-ε4* carriers, suggesting that the *APOE-ε4* allele may enhance susceptibility to HI-related cognitive decline. *APOE* plays a key role in maintaining lipid homeostasis in neurons, supporting efficient membrane synthesis, neurotransmitter release, and synaptic transmission[36]. Disruption of these processes after HI may lead to impaired neural repair and accelerated cognitive deterioration in *APOE-ε4* carriers.

Through MR analysis, we identified a causal relationship between TBI and AD. Our findings are inconsistent with those reported by Zhang et al. This inconsistency may result from the use of different AD GWAS datasets. Our study included a relatively larger number of AD cases (IGAP dataset based on 21,982 cases and 41,944 controls of European ancestry) compared with that of Zhang et al. (ieu-b-5067, including 954 cases and 487,331 controls). Therefore, we consider our results to be more reliable. Further studies are warranted to explore this causal relationship in greater detail[37]. Clarification of such a causal relationship is crucial given that TBI may be caused by cognitive impairment[38]. Compared with traditional observational studies, the use of MR analyses can provide the best evidence of causal associations by reducing the confounding effects of environmental factors and avoiding reverse causation bias[16]. However, no MR analysis had been performed to date to assess the causal relationship between TBI and AD. Our study provides direct evidence that genetically determined TBI caused a 17.2 % increase in the risk of AD. Our study found, for the first time, a causal association between TBI and AD, providing solid evidence of an increase in the lifetime risk of developing AD after TBI.

Our study had several merits. First, it involved a large cohort and a prospective design, and participants were tracked for an extended period of time, with nearly no loss to follow-up. The exposures and outcomes were clearly defined using a combination of clinical diagnoses made by professional physicians and self-reports, which are reliable in assessing HIs[39]. Comprehensive neurodegenerative events, including the diagnosis of dementia subtypes and neuroimaging findings, were analyzed as the outcomes, making it possible to assess the neurodegenerative pathways of individuals with HI/TBI. We adjusted for covariates in the models to minimize potential confounding effects. We set a buffer period of at least two years between HI/TBI and dementia diagnoses, and we performed MR analyses, ensuring that detection bias was unlikely to explain the observed associations.

However, despite these strengths, several limitations must be noted. First, Due to the lack of detailed diagnostic information for AD in the UK Biobank, we were unable to determine whether the diagnosis of AD was based on pathological confirmation. And this sample was drawn from a single country with an ethnically homogeneous population and a healthy volunteer bias, which could limit its generalizability to other groups. Additionally, the frequency of HI/TBI was low, limiting the explanatory power of the study. The heterogeneity of HI/TBI hinders research efforts and makes it difficult to precisely evaluate the true impact of HI/TBI[40]. For example, some cases of TBI are recorded as HIs, whereas others are recorded as brain injuries in the ICD10 diagnostic system[41]. Moreover, while this study was designed to establish an epidemiological link, the role of blood biomarkers jointly associated with AD and TBI, such as GFAP, warrants further investigation in future studies. As for the MR analysis, the sub-genomic statistical threshold used (*p* < 5 × 10^-6^) may have decreased the power of the conclusion.

Ultimately, this study demonstrates a long-term link between HI/TBI and an increased prevalence of dementia and degenerative changes in certain brain regions. Measures to reduce the incidence of HI/TBI and improve cognitive rehabilitation are needed to counteract the increasing burden of dementia.

## Ethics committee

The North West Multi-centre Research Ethics Committee (MREC) has granted UK Biobank certification as a Research Tissue Bank (RTB). Because of this approval, researchers can work under the RTB approval without needing an additional ethical clearance (there are certain exceptions to this which are set out in the Access Procedures, such as re-contact applications).

## Data sharing statement

The raw data of this study are available from the corresponding author upon reasonable request.

## Declaration of the use of generative AI and AI-assisted technologies in scientific writing and in figures, images and artwork

The authors declare that no generative AI or AI-assisted technologies were used in the scientific writing or in the creation of figures, images, and artwork for this manuscript.

## Fundings

This study was supported by the 10.13039/501100001809National Natural Science Foundation of China (82371434), Outstanding Youth Fund of 10.13039/501100004735Hunan Provincial Natural Science Foundation (2024JJ2097), and the Scientific Research Program of FuRong Laboratory (2024PT5108).

## CRediT authorship contribution statement

**Ziyu Ouyang:** Writing – original draft, Validation, Data curation, Conceptualization. **Bin Jiao:** Project administration. **Xuewen Xiao:** Data curation. **Qijie Yang:** Data curation. **Yuan Zhu:** Data curation. **Lu Shen:** Project administration. **Nan Li:** Writing – original draft, Validation, Data curation, Conceptualization.

## Declaration of competing interest

The authors declare that they have no known competing financial interests or personal relationships that could have appeared to influence the work reported in this paper.

## Appendix Supplementary materials

Image, application 1
