# Supplementary material for: Head injury/traumatic brain injury and the risk of dementia: An observational and Mendelian randomization study
Source: J Prev Alzheimers Dis. 2026 Jan 8;13(3):100468. doi: 10.1016/j.tjpad.2025.100468 (PMC12988369; doi:10.1016/j.tjpad.2025.100468)
Supplement: Supplementary file 1 [file mmc1.docx]

**Head Injury/Traumatic Brain Injury and the Risk of Dementia: An Observational and Mendelian Randomization Study**

***Supplementary information***

This file contains descriptions of the 13 supplementary tables to accompany the main article.

**Supplementary Figures**

**
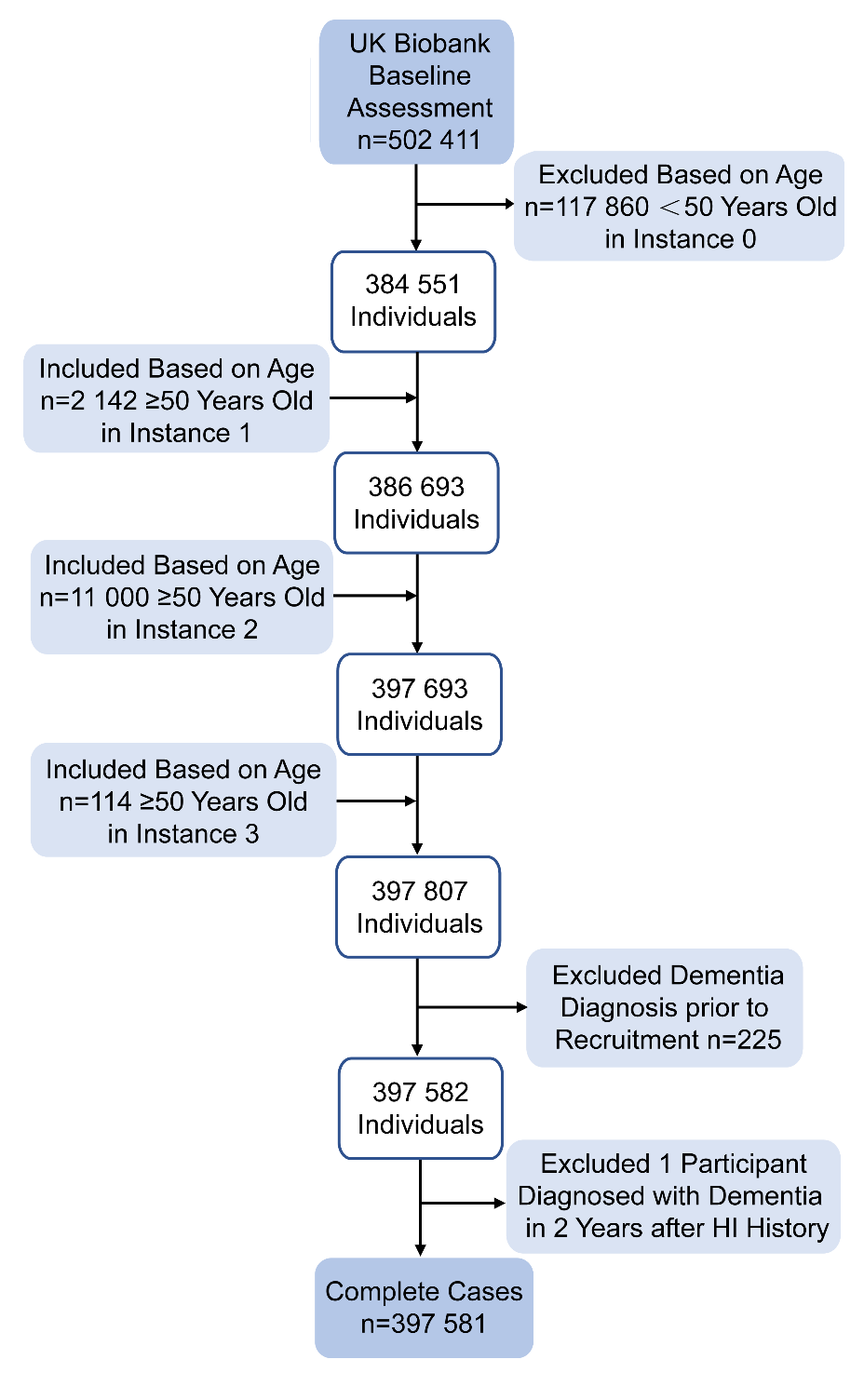
**

**Figure S1. Study Flow Chart for the Complete Case Analysis Cohort**

**Supplementary Tables**

**Table S1. Field IDs Used in the Analyses**

| 31 | Sex |
| --- | --- |
| 34 | Year of birth |
| 53 | Date of attending assessment center |
| 189 | Townsend deprivation index at recruitment |
| 190 | Reason for lost to follow-up |
| 191 | Date lost to follow-up |
| 399 | Number of incorrect matches in round |
| 738 | Average total household income before tax |
| 884 | Number of days per week of moderate physical activity for 10+ minutes |
| 1200 | Sleeplessness/insomnia |
| 1210 | Snoring |
| 1220 | Daytime dozing / sleeping (narcolepsy) |
| 1558 | Alcohol intake frequency |
| 1568 | Average weekly red wine intake |
| 1578 | Average weekly champagne plus white wine intake |
| 1588 | Average weekly beer plus cider intake |
| 1598 | Average weekly spirits intake |
| 1608 | Average weekly fortified wine intake |
| 2247 | Hearing difficulty/problems |
| 4407 | Average monthly red wine intake |
| 4418 | Average monthly champagne plus white wine intake |
| 4429 | Average monthly beer plus cider intake |
| 4440 | Average monthly spirits intake |
| 4451 | Average monthly fortified wine intake |
| 4462 | Average monthly intake of other alcoholic drinks |
| 5364 | Average weekly intake of other alcoholic drinks |
| 6138 | Qualifications |
| 10137 | Number of incorrect matches in round (pilot) |
| 10722 | Qualifications (pilot) |
| 10793 | Hearing difficulty/problems (pilot) |
| 20002 | Non-cancer illness code, self-reported |
| 20008 | Interpolated Year when non-cancer illness first diagnosed |
| 20016 | Fluid intelligence score |
| 20018 | Prospective memory result |
| 20023 | Mean time to correctly identify matches |
| 20116 | Smoking status |
| 20126 | Bipolar and major depression status |
| 20132 | Number of incorrect matches in round |
| 20191 | Fluid intelligence score |
| 21000 | Ethnic background |
| 21001 | Body mass index (BMI) |
| 25005 | Volume of grey matter (normalised to head size) |
| 25007 | Volume of white matter (normalised to head size) |
| 25009 | Volume of brain, grey+white matter (normalised to head size) |
| 25019 | Volume of hippocampus (left) |
| 25020 | Volume of hippocampus (right) |
| 25781 | Total volume of white matter hyperintensities (from T1 and T2_FLAIR images) |
| 40000 | Date of death |
| 40001 | Underlying (primary) cause of death: ICD10 |
| 40002 | Contributory (secondary) causes of death: ICD10 |
| 41270 | Diagnoses - ICD10 |
| 41271 | Diagnoses - ICD9 |
| 41280 | Date of first in-patient diagnosis - ICD10 |
| 41281 | Date of first in-patient diagnosis - ICD9 |
| 42018 | Date of the all-cause dementia report |
| 42019 | Source of the all-cause dementia report |
| 42020 | Date of Alzheimer's disease report |
| 42021 | Source of Alzheimer's disease report |
| 42022 | Date of vascular dementia report |
| 42023 | Source of vascular dementia report |
| 42040 | GP clinical event records |

**Table S2. Diagnostic Codes for Head Injury and its Subtypes**

| Head Injury | |
| --- | --- |
| ICD-9 | 800-804, 830, 850-854, 870-873, 910, 918, 921, 925, 930, 951 |
| ICD-10 | S00-S09 |
| Read Codes | XM0wZ, XM0wa, XM0w0, XM0vy, XM0vo, XE2nZ, XE2nY, XE2nd, XE2nb, XE1nK, XE1mP, XE1mO, XE1mL, XE1m7, XE1m4, XE1m2, XE1m1, XE1kV, XE1kU, XE1kT, XE1kS, XE1kR,XE1kQ, XE1kP, XaKK3, XaFxc, XaFCl, XaFCk, XaF91, XaF8m, XaEGh, XaD3B, XaB8a, XaB1Y, XaB1X, Xa9DF, Xa9DB, Xa84j, Xa7QJ, Xa7QF, Xa6Yu, Xa41k, Xa3XI, Xa3XF, Xa3By, Xa35Q, Xa35G, Xa2yc, Xa2Y9, Xa2VW, Xa2U6, Xa2TK, Xa2TJ, Xa2TI, Xa2TH, Xa2TG, Xa2TE, Xa2TC, Xa2SU, Xa2SQ, Xa2SO, Xa2Nr, Xa2Nq, Xa2Np, Xa2No, Xa2Nn, Xa2NG, Xa2NE, Xa2ND, Xa2NC, Xa2NB, Xa2NA, Xa2N4, Xa2Mb, Xa2lc, Xa2jh, Xa2IQ, Xa2IO, Xa2I9, Xa2I8, Xa2I7, Xa2I6, Xa2I5, Xa2I4, Xa2Hk, Xa2Hi, Xa2He, Xa2DE, Xa2DC, Xa2CZ, Xa2CX, Xa2CV, Xa2CT, Xa2CS, Xa2CR, Xa2Cd, Xa2Cc, Xa2Cb, Xa2Ca, Xa26E, Xa26C, XA0IE, XA0ID, XA0Ht, XA0G7, XA0G5, XA0G2, XA0G1, XA0Fz, XA0Fw, XA0FU, XA0Ft, XA0Fs, XA0FR, XA0FQ, XA0Fp, XA0Fo, XA0FL, XA0Fh, XA0Fd, XA0Fb, XA0DI, XA0Ce, XA0BL, XA0BG, XA0B9, Xa07I, XA063, XA05s, XA05R, XA05Q, XA05c, XA04L, XA049, XA048, XA03D, XA02s, XA02n, XA02m, XA02l, XA02i, XA01x, XA00V, XA00U, XA00p, XA005, XA004, X00ZY, X00Zx, X00Yr, X00Yo, X00Yn, X00Ym, X00YH, X00YF, X00TI, X00lj, X00li, X00jS, X00jQ, X00if, X00i3, X00Ed, X00dP, X00bS, X00bq, X00bb, X00aJ, X00a4, X009o, X008Z, X008X, X008W, Syu0M, Syu0G, Syu0E, Syu0C, Syu0B, Syu06, Syu04, Syu03, SK1x0, SK18., SK10x, SK109, SK106, SK105, SK104, SK103, SK102, SK101, SK100, SJ1y0, SJ17., SJ15., SJ14., SJ13., SJ12., SJ11., SJ10., SJ0z., SJ03., SJ00., SF00z, SF001, SF000, SF0.., SE1z., SE14., SE13., SE12., SE11., SE10., SE1.., SE0z., SE09., SE06., SE05., SE04., SE03., SE02., SE01., SE00., SE0.., SD8z., SD82., SD81z, SD811, SD810, SD81., SD80z, SD802, SD801, SD800, SD80., SD8.., SD0z0, SD0y2, SD0y0, SD0y., SD050, SD05., SD04z, SD042, SD040, SD04., SD030, SD022, SD020, SD02., SD012, SD011, SD010, SD01., SD00z, SD002, SD000, SD00., SD0.., S83z., S83x., S8374, S8373, S837., S8367, S8366, S8364, S8363, S8362, S8361, S8360, S836., S8354, S8352, S8351, S835., S834z, S834x, S8346, S8345, S8344, S8343, S8342, S8341, S8340, S834., S8320, S832., S831., S830., S83.., S82z., S82v0, S822., S8202, S8201, S8200, S820., S82.., S81z., S818., S817., S816., S814., S813., S812., S81.., S80z., S804., S803., S801., S800., S80.., S6z.., S64z., S647., S6460, S646., S644., S643., S640z, S6406, S6402, S6401, S6400, S640., S64.., S629., S628., S626., S624., S622., S620., S62.., S611., S610., S61.., S60z., S605., S602., S601., S600., S60.., S6..., S5y1z, S5y11, S5y1., S49G., S40z., S402., S400., S40.., S0z.., S04z., S044., S04.., S03z., S0301, S02z., S02yz, S02xz, S02x1, S02x0, S02x., S02A., S0283, S0282, S0281, S0280, S028., S027., S026., S025z, S0251, S0250, S025., S024z, S0241, S0240, S024., S023z, S0233, S023., S022z, S022x, S0228, S0225, S0224, S0223, S0222, S0221, S0220, S022., S021., S020., S02.., S01z., S0100, S010., S01.., S00z., S0003, S0000, S00.., S0..., G623., F5031 |
| TBI | |
| ICD-9 | 850-854 |
| ICD-10 | S06 |
| Read Codes | XM1OR, XE1m7, XE1m4, XE1m2, XE1m1, XaKK3, XaD3B, Xa2CR, XA0BG, XA0B9, XA04L,XA04D, XA048, XA005, X00Ed, Syu0E, S6z.., S64z., S647., S6460, S646., S644., S643., S641., S640z, S6406, S6402, S6401, S6401, S6400, S6400, S640., S640., S64.., S62A., S629., S628., S628., S626., S624., S622., S622., S620., S62.., S62.., S611., S610., S61.., S60z., S60z., S605., S605., S604., S602., S601., S601., S600., S60.., S60.., S6..., S6..., G623. |
| Mild TBI | |
| ICD-9 | 850 |
| ICD-10 | S060 |
| Read Codes | S600., S60z., S60z., S605., S605., S604., S602., S601., S601., S600., S60.. |
| Severe TBI | |
| ICD-9 | 851, 854 |
| ICD-10 | S062, S063, S065, S066 |
| Read Codes | XE1m3, XE1m1, XaKK3, XA04D, XA04C, S644., S643., S629., S628., S628., S622., S622., S620., S611., S610., S61.., G623. |

**Table S3.** **Covariate Categorizations**

*Collapsed Race Categories:*

White: British; Irish; any other white ethic background.

*College Degree or Higher Categories:*

College or university degree

Other professional qualifications, such as nursing and teaching.

Other

*Alcohol Consumption Categories:*

Low: < 14 units/week

Moderate: 14–30 units/week

High: ≥ 30 units/week

Alcohol consumption is calculated based on the self-reported number of alcohol units (equivalent to 10 mL of pure ethanol) consumed in “units per week” (for participants drinking alcohol more often than once or twice a week) or “units per month” (for participants drinking alcohol on special occasions or one to three times a month).

*Medical Comorbidity Categories:*

Hypertension

Heart/cardiac problem

Stroke

Diabetes

*Sleep Disorder Categories:*

Napping during the day

Sleeplessness / insomnia

Snoring

*Psychiatric Comorbidity Categories:*

Bipolar disorder

Major depression

**Table S4.** **Missingness for the Entire Sample**

|  | Total (N=397,581) |
| --- | --- |
| Missing race | 2,092 (0.53%) |
| Missing APOE-ε4 | 11,763 (2.96%) |
| Missing Townsend deprivation index | 433 (0.11%) |
| Missing education | 5,079 (1.28%) |
| Missing income | 65,018 (16.35%) |
| Missing alcohol consumption | 95,375 (23.99%) |
| Missing physical activity | 22,471 (5.65%) |
| Missing smoking status | 2,440 (0.61%) |
| Missing BMI | 2,747 (0.69%) |
| Missing sleep disorder | 50 (0.01%) |
| Missing hearing difficulty | 17,070 (4.29%) |

**Table S5. Baseline Demographic and Clinical Characteristics of the Participants Included in the Analyses to Determine the Association Between Diagnoses of HI and its Subtypes and Dementia Risk**

| Characteristic | | | Any Dementia (n = 7,524) | No Dementia (n = 390,057) |
| --- | --- | --- | --- | --- |
|  | | | Mean (SD) | Mean (SD) |
| Age (Years) | | | 65 (4) | 60 (5) |
| Sex (Female) | | | 3,567 (47%) | 212,449 (54%) |
| Race (White) | | | 6,899 (92%) | 357,402 (92%) |
|  | Missing | | 57 | 2,035 |
| APOE-ε4 | | |  |  |
|  | 0 risk alleles | | 529 (7.3%) | 49,247 (13%) |
|  | 1 risk allele | | 3,062 (43%) | 233,313 (62%) |
|  | 2 risk alleles | | 3,612 (50%) | 96,055 (25%) |
|  | Missing | | 321 | 11,442 |
| Townsend Deprivation Index | | | -0.95 (3.30) | -1.44 (3.02) |
|  | Missing | | 8 | 425 |
| College degree or higher | | | 2,527 (34%) | 180,114 (47%) |
|  | Missing | | 4,885 | 194 |
| Income | | |  |  |
|  | Less than £18,000 | | 2,580 (46%) | 81,604 (25%) |
|  | £18,000 to £30,999 | | 1,721 (31%) | 89,125 (27%) |
|  | £30,999 to £51,999 | | 845 (15%) | 81,304 (25%) |
|  | £52,000 to £100,000 | | 362 (6.5%) | 59,126 (18%) |
|  | £Greater than £100,000 | | 90 (1.6%) | 15,806 (4.8%) |
|  | Missing | | 1,926 | 63,092 |
| Alcohol Consumption per week | | |  |  |
|  | Non | | 48 (1.0%) | 1,354 (0.5%) |
|  | Low | | 2,302 (46%) | 133,296 (45%) |
|  | Middle | | 1,625 (32%) | 100,789 (34%) |
|  | High | | 1,054 (21%) | 61,738 (21%) |
|  | Missing | | 2,495 | 92,880 |
| Days per week of moderate physical activity | | | 3.81 (2.43) | 3.68 (2.33) |
|  | Missing | | 773 | 21,698 |
| Medical comorbidity (Yes) | | | 3,548 (47%) | 122,841 (31%) |
| Smoking Status | | |  |  |
|  | Never | | 3,470 (47%) | 207,188 (53%) |
|  | Occasionally | | 3,182 (43%) | 144,088 (37%) |
|  | Often | | 775 (10%) | 36,438 (9.4%) |
|  | Missing | | 97 | 2,343 |
| BMI (kg/m2) | | | 27.8 (4.9) | 27.5 (4.7) |
|  | Missing | | 95 | 2,652 |
| Sleep Disorder (Yes) | | | 4,186 (56%) | 221,131 (57%) |
|  | Miss | | 0 | 164 |
| Psychiatric Comorbidity (Yes) | | | 359 (4.8%) | 25,190 (6.5%) |
| Hearing difficulty (Yes) | | | 2,523 (35%) | 105,050 (28%) |
|  | Missing | | 381 | 16,689 |
| Head injury | | | 210 (2.8%) | 5,969 (1.5%) |
|  | TBI | | 65 (0.9%) | 1,713 (0.4%) |
|  |  | Mild TBI | 22 (0.3%) | 613 (0.2%) |
|  |  | Severe TBI | 32 (0.4%) | 302 (<0.1%) |
| Numbers of HI | | |  |  |
|  | None | | 7,314 (97%) | 384,088 (98%) |
|  | 1 | | 195 (2.6%) | 5,796 (1.5%) |
|  | ≥2 | | 15 (0.2%) | 173 (<0.1%) |
| Time since the First HI (Years) | | |  |  |
|  | No Injury | | 7,315 (97%) | 384,094 (98%) |
|  | 0 to ≤2.5 | | 49 (0.7%) | 1,106 (0.3%) |
|  | 2.5 to ≤5 | | 40 (0.5%) | 933 (0.2%) |
|  | 5 to ≤10 | | 52 (0.7%) | 1,558 (0.4%) |
|  | 10 to ≤20 | | 30 (0.4%) | 997 (0.3%) |
|  | 20 to ≤40 | | 22 (0.3%) | 817 (0.2%) |
|  | ＞40 | | 16 (0.2%) | 552 (0.1%) |

Abbreviation: HI, head injury; TBI, traumatic brain injury; APOE-ε4, apolipoprotein E ε4 allele.

**Table S6. Baseline Demographic and Clinical Characteristics of the Participants Included in the Analyses to Determine the Association Between Diagnoses of HI and its Subtypes and Neuroimaging Findings**

| Characteristic | | | Any Dementia (n = 90) | No Dementia (n = 42,290) |
| --- | --- | --- | --- | --- |
|  | | | Mean (SD) | Mean (SD) |
| Age (Years) | | | 62 (5) | 58 (5) |
| Sex (Female) | | | 38 (42%) | 22,285 (53%) |
| Race (White) | | | 89 (99%) | 39,047 (93%) |
|  | Missing | | 0 | 110 |
| APOE-ε4 | | |  |  |
|  | 0 risk alleles | | 10 (11%) | 5,364 (13%) |
|  | 1 risk allele | | 36 (40%) | 25,368 (62%) |
|  | 2 risk alleles | | 43 (48%) | 10,450 (25%) |
|  | Missing | | 1 | 1,108 |
| Townsend Deprivation Index | | | -1.63 (3.20) | -1.88 (2.73) |
|  | Missing | | 0 | 41 |
| College degree or higher | | | 53 (59%) | 26,581 (63%) |
|  | Missing | | 0 | 156 |
| Income | | |  |  |
|  | Less than £18,000 | | 18 (24%) | 4,529 (12%) |
|  | £18,000 to £30,999 | | 22 (29%) | 8,462 (22%) |
|  | £30,999 to £51,999 | | 19 (25%) | 11,184 (29%) |
|  | £52,000 to £100,000 | | 14 (18%) | 10,724 (28%) |
|  | £Greater than £100,000 | | 3 (3.9%) | 3,448 (9.0%) |
|  | Missing | | 14 | 3,943 |
| Alcohol Consumption per week | | |  |  |
|  | Non | | 0 (0%) | 1,354 (0.2%) |
|  | Low | | 35 (43%) | 133,296 (46%) |
|  | Middle | | 31 (38%) | 100,789 (35%) |
|  | High | | 15 (19%) | 61,738 (19%) |
|  | Missing | | 9 | 6,582 |
| Days per week of moderate physical activity | | | 3.81 (2.43) | 3.68 (2.33) |
|  | Missing | | 4 | 963 |
| Medical comorbidity (Yes) | | | 31 (34%) | 7,638 (18%) |
| Smoking Status | | |  |  |
|  | Never | | 39 (45%) | 25,734 (61%) |
|  | Occasionally | | 43 (49%) | 14,085 (33%) |
|  | Often | | 5 (5.7%) | 2,291 (5.4%) |
|  | Missing | | 3 | 180 |
| BMI (kg/m2) | | | 26.8 (4.1) | 26.7 (4.3) |
|  | Missing | | 0 | 437 |
| Sleep Disorder (Yes) | | | 55 (61%) | 23,212 (55%) |
|  | Miss | | 0 | 153 |
| Psychiatric Comorbidity (Yes) | | | 6 (6.7%) | 2,830 (6.7%) |
| Hearing difficulty (Yes) | | | 35 (41%) | 10,545 (26%) |
|  | Missing | | 4 | 1,922 |
| Head Injury | | | 2 (2.2%) | 568 (1.3%) |
|  | TBI | | 0 (0.9%) | 146 (0.4%) |
|  |  | Mild TBI | 0 (0.3%) | 56 (0.2%) |
|  |  | Severe TBI | 0 (0.4%) | 28 (<0.1%) |
| Numbers of HI | | |  |  |
|  | None | | 88 (97%) | 41,722 (99%) |
|  | 1 | | 2 (2.6%) | 562 (1.3%) |
|  | ≥2 | | 0 (0%) | 6 (<0.1%) |
| Normalized TBV | | | 1,424,362 (75,184) | 1,489,124 (73,121) |
| Normalized TGMV | | | 733,127 (50,127) | 789,300 (48,011) |
| Normalized TWMV | | | 690,858 (51,280) | 699,826 (41,010) |
| Normalized HV | | | 6,679 (1 033) | 7,631 (889) |
| WMHV | | | 12,409 (16,100) | 5,326 (6,907) |

Abbreviations: HI, head injury; TBI, traumatic brain injury; APOE-ε4, apolipoprotein E ε4 allele; TBV, total brain volume; TGMV, total grey matter volume; TWMV, total white matter volume; HV, hippocampal volume; WMHV, white matter hyperintensity volume.

**Table S7. Hazard Ratios for Dementia According to the Time Since TBI**

| Time (years) | | TBI HR (95% CI) | mTBI HR (95% CI) | sTBI HR (95% CI) |
| --- | --- | --- | --- | --- |
|  | 0 to ≤2.5 | 2.61 (1.35, 4.58) | 2.1 (1.09, 3.26) | 5.4 (2.17, 11.57) |
|  | 2.5 to ≤5 | 2.22 (1.11, 3.98) | 1.72 (0.42, 2.86) | 3.23 (1.32, 6.76) |
|  | 5 to ≤10 | 1.99 (1.07, 3.37) | 1.71 (0.42, 2.52) | 2.59 (1.51, 4.3) |
|  | 10 to ≤20 | 1.77 (0.83, 3.32) | 1.54 (0.47, 2.71) | 1.88 (0.57, 4.53) |
|  | 20 to ≤40 | 1.57 (0.82, 2.72) | 1.51 (0.45, 2.05) | 1.84 (0.55, 3.73) |
|  | ＞40 | 1.62 (0.72, 3.15) | 1.06 (0.06, 4.1) | 1.29 (0.07, 6.23) |

Hazard ratios are based on Model 2 and are adjusted for all confounders. Abbreviations: TBI, traumatic brain injury; mTBI: mild TBI; sTBI: severe TBI; CI, confidence interval.

**Table S8. Sex-Stratified Linear Regression Coefficients for HI and Dementia-Related Outcomes**

|  | Model1 | Model2 | Model3 |
| --- | --- | --- | --- |
| Male-ACD | 1.94 (1.64, 2.30) | 1.71 (1.44, 2.03) | 1.71 (1.44, 2.02) |
| Female-ACD | 1.93 (1.52, 2.44) | 1.72 (1.36, 2.18) | 1.72 (1.36, 2.18) |
| Male-AD | 1.44 (1.05, 1.97) | 1.31 (0.96, 1.79) | 1.30 (0.95, 1.78) |
| Female-AD | 1.81 (1.27, 2.58) | 1.63 (1.14, 2.33) | 1.65 (1.16, 2.36) |
| Male-VD | 2.16 (1.57, 2.98) | 1.82 (1.32, 2.52) | 1.81 (1.32, 2.50) |
| Female-VD | 1.73 (1.00, 3.00) | 1.48 (0.86, 2.57) | 1.47 (0.85, 2.55) |
| Male-TBV | -0.082 (-0.175, 0.012) | -0.08 (-0.171, 0.011) | -0.08 (-0.172, 0.011) |
| Female-TBV | -0.05 (-0.167, 0.067) | -0.038 (-0.154, 0.078) | -0.04 (-0.156, 0.076) |
| Male-TGMV | -0.038 (-0.129, 0.052) | -0.029 (-0.116, 0.058) | -0.029 (-0.116, 0.058) |
| Female-TGMV | -0.034 (-0.148, 0.081) | -0.012 (-0.125, 0.1) | -0.014 (-0.126, 0.099) |
| Male-TWMV | -0.089 (-0.191, 0.013) | -0.096 (-0.198, 0.006) | -0.097 (-0.199, 0.006) |
| Female-TWMV | -0.039 (-0.167, 0.09) | -0.041 (-0.17, 0.087) | -0.043 (-0.171, 0.086) |
| Male-HV | 0.032 (-0.067, 0.131) | 0.044 (-0.055, 0.142) | 0.045 (-0.054, 0.143) |
| Female-HV | -0.148 (-0.278, -0.018) | -0.132 (-0.261, -0.003) | -0.132 (-0.261, -0.003) |
| Male-WMHV | -0.002 (-0.103, 0.1) | -0.015 (-0.115, 0.085) | -0.016 (-0.115, 0.084) |
| Female-WMHV | -0.026 (-0.155, 0.103) | -0.037 (-0.164, 0.091) | -0.036 (-0.164, 0.091) |
| Male -Reasoning | -0.106 (-0.155, -0.057) | -0.002 (-0.048, 0.043) | -0.003 (-0.048, 0.043) |
| Female-Reasoning | -0.071 (-0.132, -0.01) | -0.043 (-0.1, 0.014) | -0.042 (-0.099, 0.016) |
| Male -RT | 0.185 (0.153, 0.216) | 0.121 (0.09, 0.152) | 0.121 (0.09, 0.152) |
| Female -RT | 0.083 (0.042, 0.124) | 0.053 (0.012, 0.094) | 0.053 (0.012, 0.093) |
| Male -PM | 0.011 (-0.044, 0.066) | -0.003 (-0.058, 0.052) | -0.003 (-0.058, 0.052) |
| Female -PM | 0.007 (-0.063, 0.077) | 0.001 (-0.068, 0.071) | 0.001 (-0.068, 0.071) |
| Male -NM | 0.005 (-0.072, 0.082) | 0.056 (-0.019, 0.132) | 0.055 (-0.02, 0.131) |
| Female -NM | 0.009 (-0.09, 0.107) | 0.027 (-0.071, 0.124) | 0.026 (-0.072, 0.124) |
| Male -TM1 | -0.006 (-0.1, 0.087) | -0.03 (-0.122, 0.062) | -0.031 (-0.123, 0.061) |
| Female -TM1 | 0.048 (-0.072, 0.168) | 0.033 (-0.087, 0.152) | 0.033 (-0.087, 0.152) |
| Male -TM2 | 0.001 (-0.092, 0.095) | -0.038 (-0.129, 0.053) | -0.038 (-0.129, 0.054) |
| Female -TM2 | 0.054 (-0.068, 0.175) | 0.031 (-0.089, 0.15) | 0.03 (-0.089, 0.15) |
| Male -SDS | -0.073 (-0.163, 0.017) | -0.025 (-0.112, 0.062) | -0.023 (-0.111, 0.064) |
| Female -SDS | -0.093 (-0.208, 0.022) | -0.071 (-0.184, 0.043) | -0.071 (-0.185, 0.043) |

Abbreviations: HI, head injury; ACD, all-cause dementia; AD, Alzheimer’s disease; VD, vascular dementia; TBV, total brain volume; TGMV, total grey matter volume; TWMV, total white matter volume; HV, hippocampal volume; WMHV, white matter hyperintensity volume; RT, reaction time; PM, pairs matching; NM, numeric memory; TM, trail making; SDS, symbol digit substitution.

**Table S9. Age-Stratified Linear Regression Coefficients for HI and Dementia-Related Outcomes**

|  | Model1 | Model2 | Model3 |
| --- | --- | --- | --- |
| ＜65-ACD | 2.44 (2.01, 2.96) | 2.05 (1.69, 2.49) | 2.03 (1.67, 2.47) |
| ≥65-ACD | 1.61 (1.33, 1.95) | 1.48 (1.22, 1.79) | 1.50 (1.23, 1.82) |
| ＜65-AD | 1.75 (1.21, 2.54) | 1.56 (1.08, 2.26) | 1.51 (1.04, 2.19) |
| ≥65-AD | 1.48 (1.09, 2.01) | 1.38 (1.02, 1.88) | 1.39 (1.02, 1.89) |
| ＜65-VD | 3.01 (2.04, 4.43) | 2.32 (1.57, 3.43) | 2.27 (1.54, 3.36) |
| ≥65-VD | 1.52 (1.02, 2.26) | 1.35 (0.90, 2.00) | 1.35 (0.91, 2.01) |
| ＜65-TBV | 0.005 (-0.08, 0.09) | -0.001 (-0.084, 0.082) | -0.001 (-0.084, 0.082) |
| ≥65-TBV | -0.357 (-0.647, -0.068) | 0.037 (-0.624, -0.048) | -0.336 (-0.624, -0.048) |
| ＜65-TGMV | 0.038 (-0.044, 0.119) | 0.037 (-0.041, 0.115) | 0.037 (-0.042, 0.115) |
| ≥65-TGMV | -0.231 (-0.51, 0.049) | -0.19 (-0.466, 0.086) | -0.19 (-0.466, 0.085) |
| ＜65-TWMV | -0.021 (-0.107, 0.065) | -0.031 (-0.116, 0.055) | -0.031 (-0.116, 0.054) |
| ≥65-TWMV | -0.285 (-0.572, 0.002) | -0.294 (-0.58, -0.007) | -0.293 (-0.579, -0.006) |
| ＜65-HV | 0.004 (-0.08, 0.088) | 0.011 (-0.072, 0.094) | 0.012 (-0.071, 0.094) |
| ≥65-HV | 0.039 (-0.248, 0.326) | 0.069 (-0.219, 0.356) | 0.064 (-0.223, 0.352) |
| ＜65-WMHV | -0.093 (-0.181, -0.006) | -0.093 (-0.178, -0.007) | -0.093 (-0.178, -0.007) |
| ≥65-WMHV | 0.245 (-0.055, 0.546) | 0.209 (-0.089, 0.507) | 0.209 (-0.09, 0.507) |
| ＜65-Reasoning | -0.075 (-0.119, -0.032) | -0.004 (-0.044, 0.036) | -0.004 (-0.044, 0.036) |
| ≥65- Reasoning | -0.119 (-0.202, -0.036) | -0.068 (-0.146, 0.011) | -0.068 (-0.146, 0.011) |
| ＜65-RT | 0.131 (0.102, 0.16) | 0.074 (0.045, 0.103) | 0.074 (0.045, 0.102) |
| ≥65-RT | 0.153 (0.1, 0.205) | 0.115 (0.063, 0.166) | 0.114 (0.063, 0.166) |
| ＜65-PM | 0.022 (-0.027, 0.071) | 0.01 (-0.039, 0.059) | 0.01 (-0.039, 0.059) |
| ≥65-PM | -0.038 (-0.129, 0.052) | -0.043 (-0.133, 0.048) | -0.042 (-0.133, 0.048) |
| ＜65-NM | 0.04 (-0.027, 0.107) | 0.075 (0.009, 0.14) | 0.074 (0.008, 0.139) |
| ≥65-NM | -0.101 (-0.252, 0.049) | -0.079 (-0.227, 0.069) | -0.082 (-0.23, 0.066) |
| ＜65-TM1 | -0.02 (-0.099, 0.06) | -0.032 (-0.111, 0.046) | -0.033 (-0.112, 0.046) |
| ≥65-TM1 | -0.029 (-0.295, 0.236) | -0.04 (-0.305, 0.225) | -0.04 (-0.305, 0.225) |
| ＜65-TM2 | 0.004 (-0.077, 0.084) | -0.02 (-0.098, 0.059) | -0.02 (-0.099, 0.058) |
| ≥65-TM2 | -0.192 (-0.465, 0.081) | -0.21 (-0.479, 0.059) | -0.205 (-0.474, 0.065) |
| ＜65-SDS | -0.023 (-0.102, 0.057) | 0.004 (-0.073, 0.081) | 0.005 (-0.072, 0.082) |
| ≥65-SDS | -0.188 (-0.454, 0.077) | -0.166 (-0.427, 0.096) | -0.171 (-0.433, 0.09) |

Abbreviations: HI, head injury; ACD, all-cause dementia; AD, Alzheimer’s disease; VD, vascular dementia; TBV, total brain volume; TGMV, total grey matter volume; TWMV, total white matter volume; HV, hippocampal volume; WMHV, white matter hyperintensity volume; RT, reaction time; PM, pairs matching; NM, numeric memory; TM, trail making; SDS, symbol digit substitution. ＜65, age less than 65 years; ≥ 65, age 65 years and older.

**Table S10.** ***APOE ε4*-Stratified Linear Regression Coefficients for HI and Dementia-Related Outcomes**

|  | Model1 | Model2 | Model3 |
| --- | --- | --- | --- |
| 0 risk allele-ACD | 1.71 (1.36, 2.11) | 1.54 (1.23, 1.91) | 1.55 (1.24, 1.92) |
| 1 risk allele-ACD | 1.95 (1.57, 2.39) | 1.69 (1.36, 2.08) | 1.69 (1.36, 2.07) |
| 2 risk allele-ACD | 1.71 (1.36, 2.11) | 1.54 (1.23, 1.91) | 1.55 (1.24, 1.92) |
| 0 risk allele-AD | 1.53 (1.1, 2.06) | 1.42 (1.03, 1.92) | 1.44 (1.04, 1.95) |
| 1 risk allele-AD | 1.41 (0.91, 2.07) | 1.26 (0.81, 1.85) | 1.25 (0.81, 1.84) |
| 2 risk allele-AD | 1.53 (1.1, 2.06) | 1.43 (1.03, 1.92) | 1.45 (1.04, 1.96) |
| 0 risk allele-VD | 1.53 (0.93, 2.35) | 1.31 (0.79, 2.02) | 1.31 (0.8, 2.03) |
| 1 risk allele-VD | 2.34 (1.58, 3.34) | 1.95 (1.3, 2.78) | 1.94 (1.3, 2.78) |
| 2 risk allele-VD | 1.53 (0.93, 2.35) | 1.3 (0.79, 2.01) | 1.3 (0.79, 2.01) |
| 0 risk allele-TBV | -0.023 (-0.162, 0.115) | -0.031 (-0.168, 0.106) | -0.035 (-0.172, 0.102) |
| 1 risk allele-TBV | -0.11 (-0.204, -0.015) | -0.1 (-0.193, -0.007) | -0.101 (-0.194, -0.008) |
| 2 risk allele- TBV | -0.023 (-0.162, 0.115) | -0.029 (-0.166, 0.108) | -0.033 (-0.17, 0.104) |
| 0 risk allele-TGMV | -0.022 (-0.151, 0.107) | -0.03 (-0.156, 0.097) | -0.062 (-0.214, 0.09) |
| 1 risk allele-TGMV | -0.063 (-0.151, 0.025) | -0.045 (-0.131, 0.04) | -0.046 (-0.132, 0.04) |
| 2 risk allele-TGMV | -0.022 (-0.151, 0.107) | -0.028 (-0.154, 0.099) | -0.033 (-0.159, 0.094) |
| 0 risk allele-TWMV | -0.026 (-0.179, 0.128) | -0.03 (-0.183, 0.123) | -0.032 (-0.184, 0.121) |
| 1 risk allele-TWMV | -0.104 (-0.209, 0) | -0.108 (-0.213, -0.004) | -0.109 (-0.213, -0.004) |
| 2 risk allele-TWMV | -0.026 (-0.179, 0.128) | -0.028 (-0.181, 0.125) | -0.03 (-0.183, 0.123) |
| 0 risk allele-HV | 0.137 (-0.011, 0.285) | 0.147 (0, 0.295) | 0.148 (0, 0.295) |
| 1 risk allele-HV | -0.1 (-0.201, 0.002) | -0.086 (-0.187, 0.015) | -0.085 (-0.186, 0.016) |
| 2 risk allele-HV | 0.137 (-0.011, 0.285) | 0.149 (0.001, 0.296) | 0.149 (0.002, 0.297) |
| 0 risk allele-WMHV | -0.062 (-0.214, 0.09) | -0.056 (-0.206, 0.094) | -0.054 (-0.204, 0.096) |
| 1 risk allele-WMHV | 0.044 (-0.06, 0.149) | 0.032 (-0.071, 0.135) | 0.032 (-0.071, 0.135) |
| 2 risk allele-WMHV | -0.062 (-0.214, 0.09) | -0.055 (-0.205, 0.095) | -0.053 (-0.203, 0.097) |
| 0 risk allele -Reasoning | -0.062 (-0.167, 0.043) | 0 (-0.098, 0.098) | 0 (-0.098, 0.098) |
| 1 risk allele - Reasoning | -0.103 (-0.152, -0.054) | -0.026 (-0.072, 0.02) | -0.026 (-0.072, 0.02) |
| 2 risk allele - Reasoning | -0.085 (-0.159, -0.011) | -0.017 (-0.086, 0.053) | -0.017 (-0.087, 0.052) |
| 0 risk allele -RT | 0.129 (0.059, 0.198) | 0.076 (0.008, 0.145) | 0.075 (0.006, 0.144) |
| 1 risk allele -RT | 0.151 (0.12, 0.183) | 0.102 (0.071, 0.134) | 0.102 (0.071, 0.134) |
| 2 risk allele -RT | 0.148 (0.099, 0.196) | 0.102 (0.054, 0.15) | 0.103 (0.055, 0.151) |
| 0 risk allele -PM | 0.102 (-0.017, 0.221) | 0.095 (-0.024, 0.215) | 0.096 (-0.023, 0.215) |
| 1 risk allele -PM | -0.013 (-0.068, 0.042) | -0.025 (-0.08, 0.031) | -0.024 (-0.079, 0.031) |
| 2 risk allele -PM | 0.016 (-0.068, 0.1) | 0.005 (-0.079, 0.089) | 0.004 (-0.08, 0.089) |
| 0 risk allele -NM | 0.069 (-0.094, 0.233) | 0.122 (-0.039, 0.282) | 0.115 (-0.045, 0.276) |
| 1 risk allele -NM | -0.068 (-0.056, 0.1) | 0.056 (-0.021, 0.133) | 0.056 (-0.021, 0.133) |
| 2 risk allele -NM | -0.068 (-0.186, 0.051) | -0.033 (-0.15, 0.084) | -0.035 (-0.152, 0.082) |
| 0 risk allele -TM1 | -0.038 (-0.238, 0.163) | -0.037 (-0.236, 0.162) | -0.035 (-0.234, 0.164) |
| 1 risk allele -TM1 | 0.027 (-0.068, 0.122) | 0.005 (-0.089, 0.1) | 0.005 (-0.09, 0.099) |
| 2 risk allele -TM1 | 0.012 (-0.13, 0.153) | -0.011 (-0.151, 0.13) | -0.009 (-0.15, 0.131) |
| 0 risk allele -TM2 | -0.063 (-0.264, 0.139) | -0.079 (-0.277, 0.118) | -0.081 (-0.279, 0.117) |
| 1 risk allele -TM2 | 0.084 (-0.013, 0.18) | 0.054 (-0.041, 0.149) | 0.054 (-0.041, 0.149) |
| 2 risk allele -TM2 | -0.068 (-0.209, 0.074) | -0.106 (-0.245, 0.033) | -0.105 (-0.244, 0.034) |
| 0 risk allele -SDS | -0.066 (-0.258, 0.126) | -0.053 (-0.241, 0.134) | -0.055 (-0.242, 0.133) |
| 1 risk allele -SDS | -0.101 (-0.193, -0.008) | -0.064 (-0.154, 0.027) | -0.063 (-0.153, 0.027) |
| 2 risk allele -SDS | -0.044 (-0.18, 0.092) | -0.003 (-0.136, 0.13) | -0.004 (-0.137, 0.129) |

Abbreviations: HI, head injury; ACD, all-cause dementia; AD, Alzheimer’s disease; VD, vascular dementia; TBV, total brain volume; TGMV, total grey matter volume; TWMV, total white matter volume; HV, hippocampal volume; WMHV, white matter hyperintensity volume; RT, reaction time; PM, pairs matching; NM, numeric memory; TM, trail making; SDS, symbol digit substitution.

**Table S11. Linear Regression Coefficients for head injury and Regional Grey Matter Volumes**

| Regional grey matter description | *β* coefficient | p value |
| --- | --- | --- |
| Volume of grey matter in Pallidum | -0.1552 | <0.0001 |
| Volume of grey matter in Temporal Pole | -0.0372 | 0.0084 |
| Volume of grey matter in Supramarginal Gyrus, posterior division | -0.1748 | 0.0167 |
| Volume of grey matter in Crus II Cerebellum | 0.0103 | 0.0187 |
| Volume of grey matter in VIIIa Cerebellum | 0.0165 | 0.0192 |
| Volume of grey matter in VIIb Cerebellum | 0.0005 | 0.0418 |
| Volume of grey matter in Middle Frontal Gyrus | -0.1593 | 0.0850 |
| Volume of grey matter in Superior Temporal Gyrus, posterior division | -0.1600 | 0.1219 |
| Volume of grey matter in Paracingulate Gyrus | -0.1643 | 0.1318 |
| Volume of grey matter in Superior Temporal Gyrus, anterior division | -0.0267 | 0.1369 |
| Volume of grey matter in Insular Cortex | -0.1813 | 0.1432 |
| Volume of grey matter in Crus I Cerebellum | -0.0469 | 0.1497 |
| Volume of grey matter in Amygdala | -0.0758 | 0.1614 |
| Volume of grey matter in Hippocampus | -0.0759 | 0.1644 |
| Volume of grey matter in Frontal Medial Cortex | -0.0367 | 0.1644 |
| Volume of grey matter in Parahippocampal Gyrus, anterior division | -0.0765 | 0.1676 |
| Volume of grey matter in Central Opercular Cortex | -0.1699 | 0.1692 |
| Volume of grey matter in Inferior Temporal Gyrus, anterior division | -0.0370 | 0.1880 |
| Volume of grey matter in Subcallosal Cortex | -0.1703 | 0.1994 |
| Volume of grey matter in Middle Temporal Gyrus, anterior division | -0.0561 | 0.2065 |
| Volume of grey matter in Lateral Occipital Cortex, superior division | -0.1337 | 0.2102 |
| Volume of grey matter in Frontal Pole | -0.1874 | 0.2105 |
| Volume of grey matter in Lingual Gyrus | -0.1901 | 0.2288 |
| Volume of grey matter in Cuneal Cortex | -0.0480 | 0.2296 |
| Volume of grey matter in Cingulate Gyrus, anterior division | -0.1070 | 0.2368 |
| Volume of grey matter in Inferior Frontal Gyrus, pars triangularis | -0.1152 | 0.2477 |
| Volume of grey matter in Postcentral Gyrus | -0.0216 | 0.2503 |
| Volume of grey matter in Caudate | -0.0575 | 0.2513 |
| Volume of grey matter in Inferior Temporal Gyrus, temporooccipital part | -0.0617 | 0.2738 |
| Volume of grey matter in V Cerebellum | -0.0593 | 0.2792 |
| Volume of grey matter in Inferior Temporal Gyrus, posterior division | -0.0526 | 0.2797 |
| Volume of grey matter in Angular Gyrus | -0.0164 | 0.2984 |
| Volume of grey matter in X Cerebellum | -0.0608 | 0.3001 |
| Volume of grey matter in Planum Polare | -0.1301 | 0.3006 |
| Volume of grey matter in Frontal Orbital Cortex | -0.1721 | 0.3097 |
| Volume of grey matter in Putamen | -0.0161 | 0.3383 |
| Volume of grey matter in Occipital Pole | -0.0939 | 0.3721 |
| Volume of grey matter in Superior Parietal Lobule | -0.0968 | 0.3793 |
| Volume of grey matter in Supracalcarine Cortex | -0.0499 | 0.3853 |
| Volume of grey matter in Precentral Gyrus | -0.0763 | 0.4011 |
| Volume of grey matter in Middle Temporal Gyrus, posterior division | -0.1202 | 0.4253 |
| Volume of grey matter in Superior Frontal Gyrus | -0.1215 | 0.4350 |
| Volume of grey matter in Juxtapositional Lobule Cortex | -0.0460 | 0.4449 |
| Volume of grey matter in Middle Temporal Gyrus, temporooccipital part | -0.1273 | 0.4457 |
| Volume of grey matter in Planum Temporale | -0.1511 | 0.4488 |
| Volume of grey matter in Temporal Fusiform Cortex, anterior division | -0.1485 | 0.4879 |
| Volume of grey matter in I-IV Cerebellum | -0.1175 | 0.5035 |
| Volume of grey matter in Thalamus | -0.1133 | 0.5382 |
| Volume of grey matter in Brain-Stem | -0.1310 | 0.5459 |
| Volume of grey matter in Frontal Operculum Cortex | -0.1185 | 0.5508 |
| Volume of grey matter in Intracalcarine Cortex | -0.0603 | 0.5626 |
| Volume of grey matter in VIIIb Cerebellum | -0.0339 | 0.5880 |
| Volume of grey matter in Ventral Striatum | -0.1323 | 0.5941 |
| Volume of grey matter in Lateral Occipital Cortex, inferior division | -0.0940 | 0.6061 |
| Volume of grey matter in Parahippocampal Gyrus, posterior division | -0.1095 | 0.6065 |
| Volume of grey matter in Precuneous Cortex | -0.0958 | 0.6105 |
| Volume of grey matter in Parietal Operculum Cortex | -0.1264 | 0.6643 |
| Volume of grey matter in Occipital Fusiform Gyrus | -0.1195 | 0.6693 |
| Volume of grey matter in Inferior Frontal Gyrus, pars opercularis | -0.0466 | 0.6928 |
| Volume of grey matter in Temporal Occipital Fusiform Cortex | -0.1129 | 0.7018 |
| Volume of grey matter in Supramarginal Gyrus, anterior division | -0.0779 | 0.7029 |
| Volume of grey matter in Cingulate Gyrus, posterior division | -0.1077 | 0.7181 |
| Volume of grey matter in Heschl's Gyrus | -0.1231 | 0.7267 |
| Volume of grey matter in Temporal Fusiform Cortex, posterior division | -0.1367 | 0.7353 |
| Volume of grey matter in VI Cerebellum | -0.1170 | 0.7585 |
| Volume of grey matter in IX Cerebellum | -0.0325 | 0.9153 |

Results from linear regression models examining the association between a history of head injury (HI) and normalized regional grey matter (GM) volumes. All regional GM volumes were normalized (z-scored) prior to analysis. The *β* coefficients represent the change in the normalized GM volume associated with a history of HI relative to the non-HI reference group.

**Table S12. Instrumental Variable in the MR Analysis Assessing the Causal Association Between TBI and AD**

| SNP | Effect_allele | Other_allele | Beta | Se | P value |
| --- | --- | --- | --- | --- | --- |
| rs76450857 | G | A | -0.0184 | 0.0367 | 0.6163 |
| rs34393279 | G | A | 0.0333 | 0.0222 | 0.1346 |
| rs780048 | G | A | -0.0325 | 0.0170 | 0.0561 |
| rs113839855 | T | G | -0.0274 | 0.0684 | 0.6892 |
| rs11144627 | G | A | -0.0439 | 0.0400 | 0.2728 |
| rs117082297 | T | C | 0.0477 | 0.0493 | 0.3329 |
| rs13267303 | C | T | -0.0103 | 0.0175 | 0.5563 |
| rs10834549 | C | T | -0.0006 | 0.0158 | 0.9719 |
| rs117841313 | C | T | -0.0744 | 0.1245 | 0.5501 |
| rs12984974 | C | A | -0.0055 | 0.0249 | 0.8255 |
| rs10974482 | G | T | 0.0137 | 0.0206 | 0.5046 |
| rs17197203 | G | A | -0.0049 | 0.0186 | 0.7925 |
| rs62106021 | G | A | 0.0349 | 0.0441 | 0.4285 |
| rs413721 | C | T | 0.0067 | 0.0164 | 0.6825 |
| rs5757512 | A | G | -0.0212 | 0.0175 | 0.2262 |
| rs11177877 | T | C | 0.0298 | 0.0165 | 0.0710 |

Abbreviations: MR, Mendelian randomization; TBI, traumatic brain injury; AD, Alzheimer’s disease
